# Developing a User-Centered Digital Clinical Decision Support App for Evidence-Based Medication Recommendations for Type 2 Diabetes Mellitus: Prototype User Testing and Validation Study

**DOI:** 10.2196/33470

**Published:** 2022-01-18

**Authors:** Kevin Larsen, Bilikis Akindele, Henry Head, Rick Evans, Purvi Mehta, Quinn Hlatky, Brendan Krause, Sydney Chen, Dominic King

**Affiliations:** 1 Center for Advanced Clinical Solution OptumHealth Optum Washington, DC United States; 2 Center for Advanced Clinical Solution OptumHealth Optum Boston, MA United States; 3 Center for Advanced Clinical Solution OptumHealth Optum Raleigh, NC United States; 4 OptumLabs UnitedHealth Group Minnetoka, MN United States

**Keywords:** clinical decision support, user-centered design, user testing, type 2 diabetes mellitus, evidence-based guidelines, validation, workflows, electronic health record, decision support, design, diabetes

## Abstract

**Background:**

Closing the gap between care recommended by evidence-based guidelines and care delivered in practice is an ongoing challenge across systems and delivery models. Clinical decision support systems (CDSSs) are widely deployed to augment clinicians in their complex decision-making processes. Despite published success stories, the poor usability of many CDSSs has contributed to fragmented workflows and alert fatigue.

**Objective:**

This study aimed to validate the application of a user-centered design (UCD) process in the development of a standards-based medication recommender for type 2 diabetes mellitus in a simulated setting. The prototype app was evaluated for effectiveness, efficiency, and user satisfaction.

**Methods:**

We conducted interviews with 8 clinical leaders with 8 rounds of iterative user testing with 2-8 prescribers in each round to inform app development. With the resulting prototype app, we conducted a validation study with 43 participants. The participants were assigned to one of two groups and completed a 2-hour remote user testing session. Both groups reviewed mock patient facts and ordered diabetes medications for the patients. The Traditional group used a mock electronic health record (EHR) for the review in Period 1 and used the prototype app in Period 2, while the Tool group used the prototype app during both time periods. The perceived cognitive load associated with task performance during each period was assessed with the National Aeronautics and Space Administration Task Load Index. Participants also completed the System Usability Scale (SUS) questionnaire and Kano Survey.

**Results:**

Average SUS scores from the questionnaire, taken at the end of 5 of the 8 user testing sessions, ranged from 68-86. The results of the validation study are as follows: percent adherence to evidence-based guidelines was greater with the use of the prototype app than with the EHR across time periods with the Traditional group (prototype app mean 96.2 vs EHR mean 72.0, *P*<.001) and between groups during Period 1 (Tool group mean 92.6 vs Traditional group mean 72.0, *P*<.001). Task completion times did not differ between groups (*P*=.23), but the Tool group completed medication ordering more quickly in Period 2 (Period 1 mean 130.7 seconds vs Period 2 mean 107.7 seconds, *P*<.001). Based on an adjusted α level owing to violation of the assumption of homogeneity of variance (*P*s>.03), there was no effect on screens viewed and on perceived cognitive load (all *P*s>.14).

**Conclusions:**

Through deployment of the UCD process, a point-of-care medication recommender app holds promise of improving adherence to evidence-based guidelines; in this case, those from the American Diabetes Association. Task-time performance suggests that with practice the T2DM app may support a more efficient ordering process for providers, and SUS scores indicate provider satisfaction with the app.

## Introduction

### Background

Diabetes affects roughly 34.2 million Americans, 90-95% of whom have type 2 diabetes mellitus (T2DM) [1]. Another 88 million adults in the United States have a condition called prediabetes, which puts them at risk for T2DM [1]. In addition to the quality-of-life challenges associated with managing the disease, T2DM can be associated with an array of complications, including kidney failure, blindness, and amputation of a toe, foot, or leg [2]. Every year, an estimated US $237 billion of the health care budget is spent on treating and managing the disease [2].

The high costs and suboptimal outcomes associated with T2DM may be associated at least in part with variability of care. For example, studies have shown that maintaining goal glycated hemoglobin (HbA_1c_) values can prevent or delay diabetes-related complications and decrease direct medical costs [2,3]. However, studies demonstrate significant variability in care paths for people diagnosed with T2DM [4], despite existing guidelines for specific lines of care; this negatively impacts their ability to achieve key health outcomes. This gap between care recommended by evidence-based guidelines and care delivered in practice is due in part to the sheer volume of information that providers must routinely digest as evidence and recommendations continually evolve.

Over the past decade, point-of-care clinical decision support systems (CDSSs) have emerged as one approach to close this gap. These systems can manifest as order sets, computerized alerts and reminders, digital guidelines, and clinical workflow tools designed to augment and support provider capabilities. Core functions of these software applications include summarizing patient facts, visualizing trends, supporting documentation, generating reminders, and making therapy recommendations to the provider [5,6].

CDSSs generally support the provider by (1) pulling together relevant patient facts in a manner that is efficiently assessed, and (2) bringing up-to-date, evidence-based clinical guidelines to the point of care where clinical decisions are made. Sutton et al [5] identified benefits of adoption along with risks that should be mitigated through strategic design. For example, a CDSS may elevate adherence to clinical guidelines, but it may risk creating excessive trust in the system without appropriate checks. Improved retrieval and presentation of patient data through a CDSS may support better choices in treatment, but it also risks disrupting existing workflow if usability is not adequately evaluated. Determining the success of clinical decision support (CDS) tools ultimately depends on measurable improvements in the quality of care. The literature provides examples of improvements in process-related and clinical outcomes [4]. For example, in randomized trials, CDS interventions have been associated with increased hemoglobin testing rates and with steeper declines in measured HbA_1c_ levels—an indication of glycemic control [6-9].

Measurable quality-of-care improvements are dependent on good CDSS design. Sim et al [10] developed and tested a web-based CDS tool, a diabetes dashboard that provided graphic summarization of laboratory results and was intended to facilitate the interpretation of results and flag tests needed by the patients. User testing demonstrated performance advantages over the electronic health record (EHR) for recognition of abnormal test results, identification of long-term trends, and awareness of which tests were due for repeating. However, participants using the dashboard were not able to better determine whether a treatment adjustment was required. The failure to find a treatment-decision benefit should not be unexpected given that the dashboard was not designed to make patient-specific treatment recommendations. The outcome highlights the need for an interface that supports the processing of patient facts and leverages evidence-based guidelines.

### Objectives

The aim of this study was to evaluate the application of a user-centered design (UCD) process toward the development of a prototype software that serves as a medication recommender for T2DM (the T2DM app). The prototype T2DM app is a standards-based CDS tool that provides evidence-based medication recommendations to health care providers with the aim of improving adherence to the latest evidence-based clinical guidance and reducing cognitive load for clinicians making prescribing decisions. The prototype T2DM app integrates into existing EHR systems that clinicians use as part of standard practice to review patient records, order tests and prescriptions, etc.

## Methods

### Methods Overview

The prototype app development consisted of two phases: (1) a predevelopment analytic phase to learn about user needs, the context of use, and specific workflow associated with reviewing patient facts and ordering medications; and (2) iterative user testing of the prototype app itself. The validation study with the final version of the prototype app addressed the following research questions: (1) is medication ordering with the T2DM app associated with more medication orders that align with American Diabetes Association (ADA) evidence–based guidelines compared to ordering medications with a typical EHR? (2) Is medication ordering with the T2DM app associated with faster overall task times compared to ordering medications with a typical EHR? (3) Is medication ordering with the T2DM app associated with lower perceived cognitive load (as measured by the National Aeronautics and Space Administration Task Load Index [NASA TLX]) compared to ordering medications with a typical EHR?

### Prototype App Development

#### Analytic Phase

The analytic work that provided the foundation for the first interactive prototype entailed the review of ADA guidelines [11] to determine priorities for the selection of patient facts that would need to be pulled into the T2DM app from the EHR. The team also conducted interviews with 8 conveniently selected clinical leaders from care delivery organizations of significant scale to learn about the intended users, the context of use, and the specific workflow in their EHR associated with reviewing patient facts and ordering medications to manage T2DM.

#### User Testing Phase

Once the initial prototype was developed on the basis of findings from the analytic phase, 8 iterative rounds of user testing were carried out to get feedback on different parts of its evolving design. These were conducted remotely from May 4 to October 2, 2020, owing to COVID-19 restrictions, and each session was recorded. For each round of user feedback, a sample of prescribers were included in 1-hour, one-on-one test sessions with the prototype app. In total, 16 participants were recruited directly from a single large provider network, and the remaining participants were recruited via a national third-party recruitment service. The latter were compensated at the prevailing market rate for physicians, physician assistants, and nurse practitioners. In total, 25 MD physicians, 11 nurse practitioners, and 15 physician assistants participated in the user testing. We used a talk-aloud method for data collection while prescribers reviewed and ordered medications with the prototype.

In addition, during 5 of the 8 rounds of user testing, participants completed the System Usability Scale (SUS) questionnaire at the end of their sessions (for logistical reasons [sample size and participant time], the SUS was not administered in rounds 5, 7, and 8). The SUS questionnaire is an industry standard for evaluating the usability of software applications consisting of 10 statements with 5 response options (ranging from “Strongly Disagree” to “Strongly Agree”) to each question. The statements are as follows:

- I think that I would like to use this system frequently.
- I found the system unnecessarily complex.
- I thought the system was easy to use.
- I think that I would need the support of a technical person to be able to use this system.
- I found the various functions in the system were well integrated.
- I thought there was too much inconsistency in this system.
- I would imagine that most people would learn to use this system very quickly.
- I found the system very cumbersome to use.
- I felt very confident using the system.
- I needed to learn a lot of things before I could get going with this system.

The SUS survey yields a single number that represents a composite measure of the overall perceived usability of the system. SUS scores have a range of 0 to 100 and the score is a relative benchmark that is used against other iterations of the system. The SUS is a reliable and valid measure of system satisfaction. Sauro [12] reports that the average SUS score from 500 studies across various products (eg, websites, cellphones, and enterprise systems) and across different industries is 68. A SUS score above 68 is considered above average and anything below 68 is below average.

### Validation Study

#### Participants

In total, 43 participants completed the 2-hour remote evaluation study of the prototype T2DM app that was developed and refined through the user testing phase. Participants were recruited via a national third-party recruitment service. The study population included 21 MD and 22 non-MD physicians (5 nurse practitioners, 5 physician assistants, 6 nurses, and 6 pharmacists). To be included in the study, candidates were required to (1) have at least 1 year of experience treating T2DM, preferably in a family or internal medicine practice; (2) currently prescribe, prescribe on behalf of, or provide medication recommendations as part of their current role; and (3) currently interact with 15 or more patients per day. Participant compensation was set at the prevailing market rate for physician assistants, nurse practitioners, pharmacists, and nurses.

Medical and legal review were conducted to ensure no aspect of clinical or legal regulations or ethical considerations were overlooked. Formal institutional review board or ethical review was not required in the study because no protected health information was included, and participation was limited to usability testing and providing feedback about the app. No private information about participants was collected or included in the analysis and study. All participants gave verbal consent and were reimbursed for their time.

A Universal Design framework was followed for accessibility and to accommodate a wide range of people (eg, people with color blindness), and care was taken to convert typical in-person interaction to virtual to avoid exposure to COVID-19.

#### Stimuli

**Figure 1:**
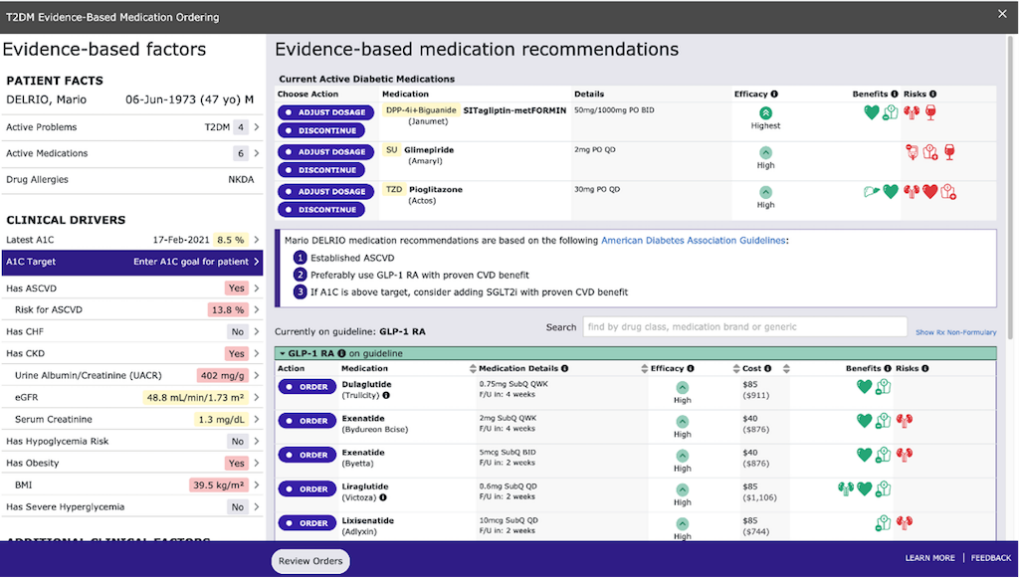
A screenshot of the prototype T2DM app. A1C: glycated hemoglobin, ASCVD: atherosclerotic cardiovascular disease, CHF: congestive heart failure, CKD: chronic kidney disease, eGFR: estimated glomerular filtration rate.

**Figure 2:**
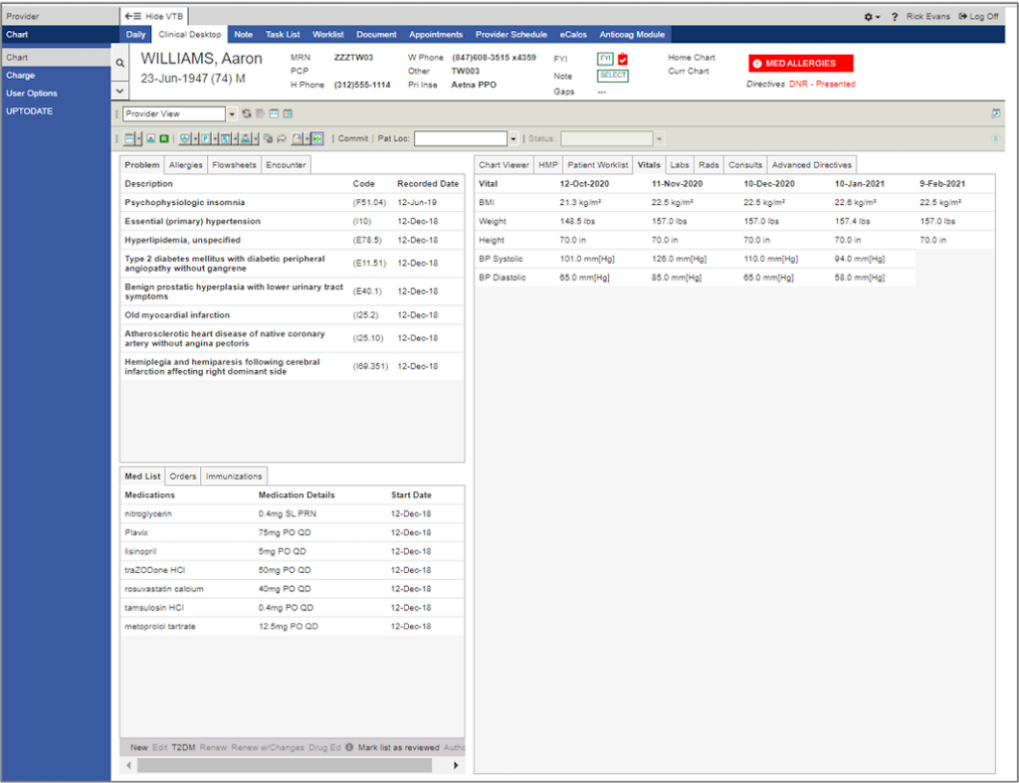
A screenshot of the mock electronic health record. BP: blood pressure.

**Figure 3:**
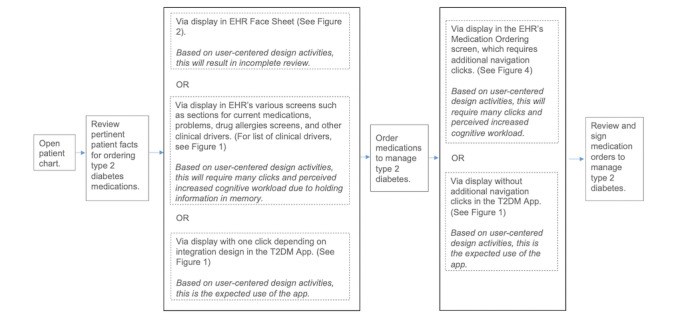
Schematic of workflow to complete an ordering task in an EHR that includes the integrated prototype T2DM app. EHR: electronic health record, T2DM: type 2 diabetes mellitus.

**Figure 4:**
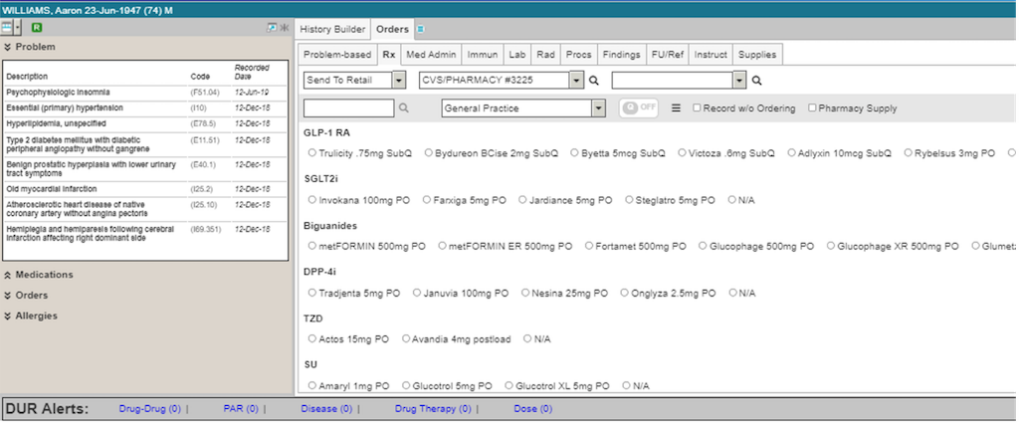
Mock medication ordering screen in the electronic health record. DPP-4i: dipeptidyl peptidase 4 inhibitor, GLP-1RA: glucagon-like peptide-1 receptor agonist, SGLT2i: sodium/glucose cotransporter-2 inhibitor, SU: sulfonylurea, TZD: thiazolidinedione.

The prototype T2DM app (Figure 1) pulls data from the EHR to present patient information in a user-friendly way on the left side of the screen. The right side of the screen displays a list of current diabetes medications, evidence-based recommendations based on the latest ADA guidelines, and a table of on- and off-guideline medications for ordering. In addition to the prototype T2DM app, a second prototype that is a close representation of a commercial EHR user interface was developed for use in the validation study (Figure 2). Both prototypes (the mock EHR and the T2DM) presented mock patient facts that simulate patients with T2DM. The workflow was captured as each participant interacted with both prototypes (Figures 3 and 4).

#### Apparatus and Test Environment

The study was conducted between March 15 and 19, 2021, and because of continued COVID-19 social distancing restrictions, the test sessions were conducted remotely via a usability testing platform (Loop11) or via a web conference tool (Zoom or WebEx). One independent moderator was present with the participant throughout the session. Several sessions were observed by members of the product development team; two of the observing team members and the moderator asked participants clarifying questions during the session. The participants employed their own computers to display and interact with the apps. Although the environment with respect to seating, lighting, sound levels, temperature, and humidity varied, participants were most generally seated at a table or desk in a personal space within their home. In most cases, the participants shared their screens and interacted with the apps on their own desktops. In several cases, the moderator shared their screen and passed control of mouse and keyboard to the participant.

The method of data capture depended on the platform employed in the session. The Loop11 platform captured videos, task time, and screen review data; the web conference tools captured video only. In all cases, a notetaker recorded participant responses, and medication orders were collected manually from screen shots taken during the time of the study and determined by video review.

Participants also completed the Kano Model Survey. The Kano Model is a tool that can be used to prioritize the *critical to quality* characteristics, as defined by the *voice of the customer* [13]. The three categories identified by the model are as follows: (1) *Must-Have*: whatever the quality characteristic is, it must be present, such that if it is not, the customer will go elsewhere! (2) *Linear or Performance*: the better we are at meeting these needs, the happier the customer is. (3) *Exciter or Delighter*: those qualities that the customer was not expecting but received as a bonus.

The task data collected included “pass” or “fail” (medication order adheres or does not adhere to evidence-based recommendations), task time, subjective comments from the Kano Model Survey, and video recordings of the computer screen and audio.

#### Study Design

**Figure 5:**
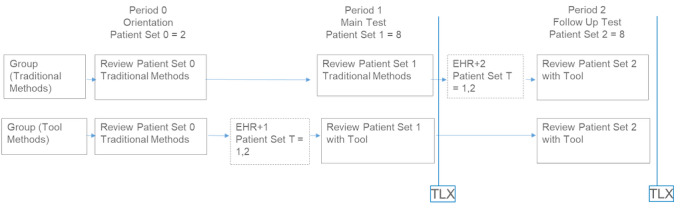
Schematic of the study design. EHR: electronic health record, TLX: Task Load Index.

The study employed a 2 (Group) × 2 (Time Period) mixed model simulated-use design (shown in Figure 5). Participants were randomly assigned to one of two groups (Traditional, Tool) with the constraint that each group was comprised of 50% MDs and 50% other medical licenses. Participants in both groups were asked to review two sets of eight mock patients and order diabetes medications. Participants in the Traditional group employed the mock EHR for reviewing and ordering in Period 1 and employed the prototype T2DM app for reviewing and ordering in Period 2. Participants in the Tool group employed the prototype T2DM app in both time periods. Both groups were afforded the opportunity to access any online resources that are typically used as part of their medication decision-making (eg, websites such as UpToDate, ADA evidence-based guidelines).

Each reviewing and ordering period was followed by administration of the NASA TLX to measure the perceived cognitive load associated with that performance period [14]. The NASA-TLX solicits ratings for mental demand, physical demand, temporal demand, performance, effort, and frustration on a scale from “Very Low” to “Very High.” The web-based interface presented the scale as 10 radio buttons that were not numbered; responses were coded 0-100 consistent with extant literature [14]. Pairwise rankings of the indices were collected to generate weights for computation of the TLX score.

The data were analyzed to test the hypothesis that providers would more frequently align their orders with the ADA evidence-based guidelines when using the T2DM app, and that the process would be more efficient and less taxing. The hypothesis predicts performance advantages for the Tool group during the first period, and a greater change in duration for Traditional group in Period 2.

#### Procedure

Each 2-hour session was conducted individually and began with guidance about the resolution of technical difficulties associated with the employed platforms and the informed consent process. The overall study process for both participant groups is shown in Figure 3.

##### Period 0

Orientation of participants to the EHR prototype. The moderator walked participants through the user interface and then participants completed 2 practice trials reviewing mock patient facts and ordering diabetes medications using traditional EHR methods. The purpose of the orientation was to ensure that participants understood the task. Both groups experienced the same protocol for this period.

##### Training Period

Orientation to the prototype T2DM app. The moderator walked participants through the user interface and then participants completed two practice trials reviewing mock patient facts and ordering diabetes medications using the prototype T2DM app. The Tool group experienced this protocol immediately before Period 1; the Traditional group experienced this protocol immediately before Period 2.

##### Period 1

Participants reviewed a set of 8 mock patients and ordered diabetes medications on the basis of each mock patient’s facts. Participants were instructed to complete each order within 5 minutes and to do so as though they were at work. Participants in the Traditional group employed the mock EHR during this period; participants in the Tool group employed the prototype T2DM app.

##### TLX 1

Participants completed the NASA TLX survey aimed to measure perceived mental cognitive load for the tasks completed in Period 1.

##### Period 2

Participants reviewed another set of 8 mock patients and ordered diabetes medications on the basis of each mock patient’s facts. As in period 1, participants were instructed to complete each order within 5 minutes and to do so as though they were at work. Participants in both groups employed the prototype T2DM app.

##### TLX 2

Participants completed the NASA TLX survey aimed to measure perceived cognitive load for the tasks completed in Period 2.

#### Data Analysis

We analyzed data using SPSS (version 25.0; IBM Corp). Dependent variables in the study (percent adherence to evidence-based guidelines, task time, screens reviewed, and perceived workload rating) were all subjected to a 2 × 2 mixed model analysis of variance (ANOVA) with Period (Period 1 vs Period 2) as the within-subjects factor and Group (Traditional vs Tool) as the between-subjects factor. Statistical significance was accepted at a level of *P*<.05.

## Results

### Prototype App Development

#### User Testing

Each round of user testing focused on different elements of the prototype app while also iteratively reviewing changes that resulted from the previous round or rounds. Changes became smaller and more focused with each successive round of testing until the final version was reached after round 8. Table 1 provides information about each round.

**Table 1:** Focus and results for each round of user testing.

Round	User testing focus	Changes informed by results
1	What clinical information prescribers used to inform medication ordering, medication ordering workflow, and visual presentation of information on the left side of the screen	Patient fact details, medication ordering workflow, and user interface improvements
2	What additional clinical information prescribers needed to inform diabetes medication ordering, details of the patient facts, and on and off guidelines for evidence-based prescribing	Patient facts details, order summary screen, and medication details relevant to prescribers
3	Adequacy of clinical information for prescribers to make appropriate medication decisions	Patient fact details, on and off guideline medication details, and order summary screen
4	Importance of various features for prescribers (eg, patient facts, clinical drivers, on and off guideline evidence–based medication table, and ordering)	Prioritization of included features
5	Ease of use for finding patient facts and ordering medication	User workflow and interface design
6	Ease of use for updated interface design	User workflow and interface design
7	Ease of use for finding patient facts and ordering or discontinuing medication	Ordering and discontinuing workflow
8	Utility and clarity of app user guide and product information	Presentation of information in user guide and product information

#### Subjective Comments

Comments made by user testing participants (Table 2) were used in determining design changes for each round.

**Table 2:** Sample comments from user testing.

Context	Comments
On laboratory results and interpretation	“I’m the one with the medical degree, not the computer. I need to know where things are coming from.” [estimated glomerular filtration rate finding]
Presentation of clinical drivers	“It’s amazing…I really like the way it pulls clinical drivers into one location so you can drive your recommendations based on that.” “I'm not necessarily going to trust an app to be the end goal. If it has an explanation, I might have a little more trust.”
Presentation of medication cost	“Cost should be specific to the patient’s insurance in order to be useful”
Flagging allergies	“You need to know that [allergies] if you are looking at medications…. I would want that {allergies}to be more prominent.”
Drug utilization review checking in electronic medical records	”I didn’t realize it would take me to the EMR, I thought I would be able to do it through the app.”

#### SUS

The SUS scores were “average” or “above average” on each of the rounds of user testing where measured (Table 3). While the averages for rounds 1-4 were all above 68, the lower average for round 6 resulted from using an alternate design that participants perceived as less user-friendly; this design was subsequently abandoned in favor of the earlier design.

**Table 3:** System Usability Scale scores for 5 of the 8 rounds of user testing.

Round	Average score	Score range	Scores, n (individual scores)
1	83	60-95	7 (80, 87.5, 87.5, 87.5, 82.5, 60, 95)
2	73	65-78	6 (75, 65, 77.5, 72.5, 75, 75)
3	86	63-100	8 (95, 80, 62.5, 90, 97.5, 100, 85, 75)
4	81	63-95	6 (72.5, 87.5, 77.5, 95, 62.5, 90)
6	68	50-78	5 (50, 60, 90, 60, 77.5)

### Validation Study

#### Combined Kano Model Study Results

**Figure 6:**
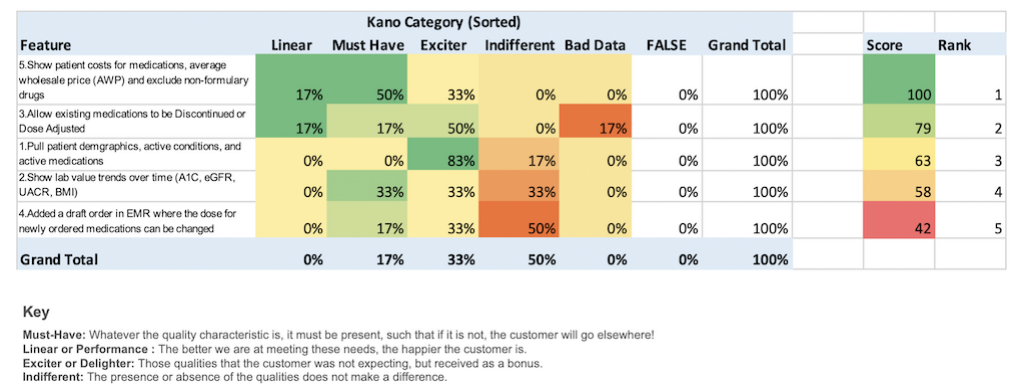
Kano Model Survey results. A1C: glycated hemoglobin, eGFR: estimated glomerular filtration rate, EMR: electronic medical record, UACR: urine albumin-creatinine ratio.

In total, 14 of the validation study participants responded to the Kano Model Survey (Figure 6).

#### Alignment With Evidence-Based Guidelines

Orders were scored as *pass* if the participant ordered any medications that were on-guideline, discontinued any medications not aligned with guidelines (ie, a recommended discontinuation) or if the guidelines recommended against medication changes, and *fail* if any ordered medication was off-guideline.

**Figure 7:**
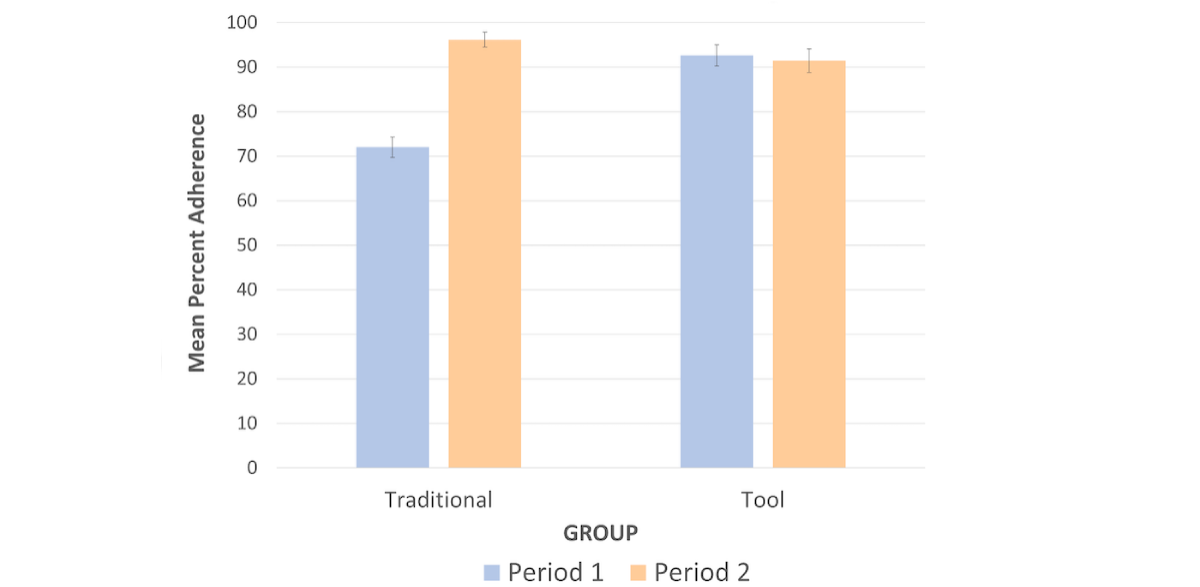
Adherence to American Diabetes Association evidence-based guidelines.

The mean percentage adherence to ADA evidence-based guidelines is shown in Figure 7. There was a main effect of Group on adherence to guidelines (*F*_1,41_=8.99, *P*=.005, 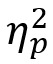
=0.18). As hypothesized, medication ordering by the Tool group was more frequently aligned with guidelines (mean 92.1) than it was by the Traditional group (mean 84.2). Furthermore, there was a main effect of Period on adherence to guidelines (*F*_1,41_=37.63, *P*<.001, 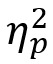
=0.48). Medication ordering in Period 2 was more frequently aligned with guidelines (mean 93.7) than it was during Period 1 (mean 82.6).

However, there was a significant Group × Period interaction (*F*_1,41_=45.43, *P*<.001, 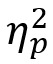
=0.53). For the Traditional group, ordering was more aligned with guidelines during Period 2, when the T2DM Tool was first introduced (mean 96.2), than it was in Period 1 (mean 72.0; *t*_20_=–8.47, *P*<.001). For the Tool group, ordering was similarly aligned to guidelines during Period 1 (mean 92.6) and Period 2 (mean 91.5; *t*_21_=0.46, *P*=.65).

Of particular interest was a planned comparison between groups during Period 1. As hypothesized, medication ordering by the group using the prototype T2DM app was more frequently aligned to ADA evidence–based guidelines (mean 92.6) than medication ordering was by the group using the mock EHR (mean 72.0; *t*_41_=–6.20, *P*<.001).

#### Task Time

Measurement of task times were impacted by technology issues (ie, poor internet connections), which resulted in extreme task times. Extreme task times were excluded based on a cutoff of 3 SD. Task time SDs were calculated using all available task time data. Task times greater than (and less than 3 SD) were excluded from the data analysis (ie, from the computation of each participant’s mean for each period). The percentage of trials removed by the 3 SD trim was 1.8%. There were no task times 3 SD below the mean because that was a negative value.

**Figure 8:**
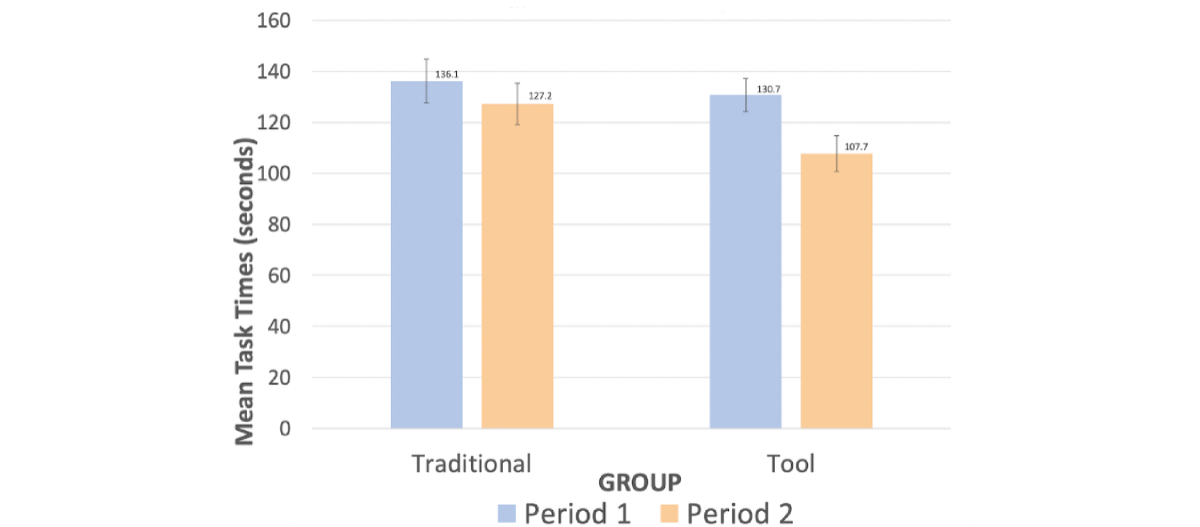
Task times during prescribing.

The mean task time used for the completion of orders is shown in Figure 8. There was no main effect of Group on task time (*F*_1,41_=1.46, *P*=.23, 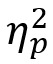
=0.03), but there was a main effect of Period (*F*_1,41_=26.70, *P*<.001, 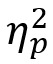
=0.39). Medication ordering time was lower during Period 2 (mean 117.2) than it was in Period 1 (mean 133.4).

There was also a significant Group × Period interaction (*F*_1,41_=5.23, *P*=.03, 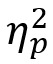
=0.11). The Tool group completed prescription orders more quickly during the second period (mean 107.7) than the first period (mean 130.7; *t*_21_=6.49, *P*<.001). For the Traditional group, task time did not significantly differ between Periods 1 (mean 136.1) and Period 2 (mean 127.2; *t*_20_=1.74, *P*=.10). Finally, Period 1 task times also did not significantly differ between the two groups (*t*_41_=0.50, *P*=.62).

#### Screens Reviewed During Prescribing

For technical reasons, screen review data was not captured for 9 participants from the Traditional group and 5 participants from the Tool group.

There was no main effect of Group on the number of screens reviewed (*F*_1,26_=0.51, *P*=.48, 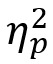
=0.02), and there was no main effect of Period (*F*_1,26_=0.73, *P*=.40, 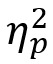
=0.03). Because the variance was found to be unequal across groups (by Box M and Levene tests), to reduce the probability of type 1 error, the α level was adjusted accordingly to be increasingly conservative (α’=.001). Based on the adjusted α level, there was no Group × Period interaction effect (*F*_1,26_=7.16, *P*=.01, 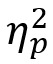
=0.22).

#### Perceived Cognitive Load (NASA TLX)

Of the 43 participants, 9 participants from the Traditional group and 6 participants from the Tool group were not able to complete the survey for logistical or technical reasons.

There was no main effect of Group on the NASA TLX score (*F*_1,34_=2.30, *P*=.14, 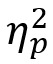
=0.06), and there was no main effect of Period (*F*_1,34_=2.13, *P*=.15, 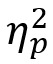
=0.06). There was no significant Group × Period interaction (*F*_1,34_=0.23, *P*=.64, 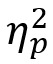
=0.01). Finally, the perceived workload associated with Period 1 did not significantly differ between the two groups (*t*_34_=1.14, *P*=.26).

#### Effect of Provider Type

As part of our analysis, we attempted to evaluate the effect of provider type on the studies using mixed ANOVAs with the clinical role (MD vs non-MD physicians) between subjects and test period (Period 1 and Period 2) within subjects. There was no significant main effect of clinical role on adherence to guidelines (*F*_1,41_=4.05, *P*=.05, 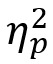
=0.09), and role × time interaction (*F*_1,41_=1.87, *P*=.18, 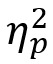
=0.04). Furthermore, there was no significant main effect of clinical role on test period (*F*_1,41_=3.86, *P*=.06, 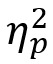
=0.09), and role × time interaction (*F*_1,41_=.79, *P*=.38, 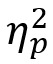
=0.02). Provider type analysis was not conducted for other parts of the study, partly owing to the sample size and initial findings.

## Discussion

### App Effectiveness, Efficiency, and User Satisfaction

This applied study aimed to develop a prototype medication recommender (T2DM) app via a thorough UCD process and evaluate the design for effectiveness, efficiency, and user satisfaction. The prototype T2DM app is considered *effective* if it supported care decisions that better aligned with evidence-based guidelines and more *efficient* as measured by reduced task time or reduced cognitive load for participants.

For the primary research question of whether medication ordering with the prototype T2DM app would be more frequently aligned to ADA evidence–based guidelines than medication ordering without the app and using the mock EHR (ie, app effectiveness), the prototype T2DM app proved effective when measured within as well as between participants. Providers who ordered medications using the mock EHR first became more aligned to ADA guidelines when they switched to the prototype T2DM app. When comparing the method employed in the first period, the group of providers that used the T2DM app were more aligned to ADA guidelines than the group that used the mock EHR.

For the primary research question of whether medication ordering with the prototype T2DM app would be accomplished more quickly than that without the app (with the mock EHR; ie, app efficiency): medication ordering was accomplished more quickly in the second period, which can reasonably be expected owing to practice. When comparing methods employed in the first period, the group of providers that used the prototype T2DM app did not complete their medication ordering more quickly than the group that used the mock EHR. However, the group that used the prototype T2DM app in both time periods improved more during the second period than the group that switched from the mock EHR to the prototype T2DM app. This outcome suggests that providers may be more efficient when using the T2DM app, but not until they have become more familiarized with its display and features. The greater efficiency in the second period for the tool-only group compared to the EHR group is likely owing to the inherent design and features of the prototype app: it is fit-for-purpose, avoids navigation distractions in the chart, and pulls information from different parts of the EHR to prefill the clinical facts. There was also no significant effect of the difference in provider type in alignment to guidelines or task times.

For the primary research question of whether medication ordering with the prototype T2DM app would be accomplished with lower perceived cognitive load than that without the app (with the mock EHR; ie, app efficiency), NASA TLX scores were generally low. Although benchmarking is not possible with the TLX, a published analysis of 237 studies would place our overall mean of 42.5 at approximately the 35th percentile [15]. Clearly, medication ordering was not exceptionally taxing with either interface, but the failure to find differences should not be interpreted as a floor effect. Despite changes in ordering behavior, the perceived cognitive load was relatively stable.

Finally, the prototype T2DM app was evaluated for *user satisfaction* throughout the iterative rounds of user testing. SUS scores throughout the iterative design process were at or above 68, which is considered average across industries. Round 6, the last iterative user testing during which the SUS was administered, had an average SUS score of 68. This was the lowest average SUS score obtained during the design process. The design evaluated in Round 6 went against several good user interface principles, and the prototype was not as interactive as the previous prototypes; hence, participants were not able to experience many of the previously identified valuable features. SUS scores for EHR systems have been shown to be lower than the average (68) from across different industries [12]. Melnick et al [16] benchmarked EHR usability by having 870 physicians complete the SUS questionnaire on the basis of their experiences with their own EHR system. The mean SUS score was 45, a score characterized as not acceptable and given a grade of F. Thus, although we caution against making direct comparisons, these data provide a favorable background for considering the usability of the prototype T2DM app.

The information from this prototype testing will contribute to the development of a real CDS-T2DM app, which will leverage SMART (Substitutable Medical Applications and Reusable Technologies) on FHIR (Fast Healthcare Interoperability Resource) technology. It is important to note that clinical decision support apps may be subject to FDA (Food and Drug Administration) review and approval. The scope of FDA oversight and compliance with any related regulatory requirements was beyond the scope of this study on the prototype app.

### Limitations

We acknowledge some limitations of our study, including (1) a small sample size, (2) participant attrition in certain rounds of the study and NASA TLX assessment, and (3) difference in platforms for observation (The Loop11 platform captured videos, task time and screen review data; the web conference tools captured video only). Furthermore, owing to the limited sample size, we were neither able to reliably assess the effect of provider type (MD vs non-MD physicians) across the studies nor the impact of speed to adoption on task time. We believe that additional research is needed to further evaluate the effectiveness and efficiency of the T2DM app as it launches and becomes more widely used.

### Conclusions

T2DM and prediabetes affect millions of Americans, often resulting in harmful complications, additional chronic conditions, disability, premature death, and significant patient and system costs. Adherence to clinical treatment guidelines can improve patient health outcomes and reduce patient and system costs. Complexity of medical guidelines combined with limitations on providers’ time can impede guideline adherence. While CDSSs can help, lack of user involvement in their development can further impede progress and result in mistrust in technology solutions.

Through deployment of the UCD process, we developed a prototype of a medication recommender app that promises to improve adherence to ADA evidence–based guidelines and support a more efficient and user-friendly ordering process for the provider in the management of T2DM. CDSSs offer promising solutions for closing the gap between provider behavior and evidence-based practice, and this study suggests that realizing their full promise may depend on greater attention to design from a user-centered perspective. Such a process could be beneficial in developing effective CDSSs for other conditions and tasks with associated improvement in quality and costs for patients, providers, and the health care system.

## Abbreviations

ADA: American Diabetes Association
ANOVA: analysis of variance
CDS: clinical decision support
CDSS: clinical decision support system
EHR: electronic health record
FDA: Food and Drug Administration
FHIR: Fast Healthcare Interoperability Resource
HbA_1c_: glycated hemoglobin
NASA TLX: National Aeronautics and Space Administration Task Load Index
SMART: Substitutable Medical Applications and Reusable Technologies
SUS: System Usability Scale
T2DM: type 2 diabetes mellitus
UCD: user-centered design
